# Mindful Eating, Disordered Eating, and Exercise Addiction: A Bayesian Gaussian Network Analysis

**DOI:** 10.3390/nu18152524

**Published:** 2026-08-04

**Authors:** Merve Rumeysa Alpay, Cemal Berkan Alpay, Attila Szabo

**Affiliations:** 1 Faculty of Sport Science, Osmaniye Korkut Ata University, Osmaniye 80000, Türkiye; 2 Department of Sport Science, Ağrı İbrahim Çeçen University, Ağrı 04100, Türkiye; cbalpay@agri.edu.tr; 3 Faculty of Health and Sport Sciences, Széchenyi István University, 9026 Győr, Hungary

**Keywords:** Bayesian Gaussian network modeling, bulimic tendencies, eating regulation, recreational exercisers, mindful eating, risk behavior

## Abstract

**Objectives:** This study examined component-level relationships among mindful eating, disordered eating attitudes, and risk of exercise addiction (REA) in recreational exercisers. Using Bayesian Gaussian network analysis, the study aimed to identify conditional associations and central variables across these constructs. **Methods:** Data were collected from 303 regular exercisers in Türkiye aged 18–57 years (M = 24.42, SD = 7.10; 57.8% male, 42.2% female). Participants completed the Eating Attitudes Test-26 (EAT-26), the Mindful Eating Questionnaire (MEQ), and the Expanded Exercise Addiction Inventory-3 (EAI-3), with all subscales entered as nodes in a 12-node Bayesian Gaussian network. The estimated network contained 22 nonzero edges among 66 possible connections (sparsity = 0.667), indicating a moderately sparse structure. The robustness of the recovered structure was examined against a regularized frequentist counterpart and against models adjusting for age, sex, and exercise frequency/duration. **Results:** The strongest conditional associations occurred within rather than between instruments: EAT-26 Restriction (Diet/Calorie Control) and EAT-26 Bulimia and Food Preoccupation, EAI-3 Healthy Relevance and EAI-3 Addiction Tendency, MEQ Eating without Thinking and MEQ Emotional Eating, and EAT-26 Bulimia and Food Preoccupation and EAT-26 Social Pressure. Cross-domain associations were present but consistently weaker, with the mindful-eating and exercise-addiction domains bridged principally by MEQ Eating Discipline–EAI-3 Healthy Relevance and MEQ Conscious Eating–EAI-3 Addiction Tendency. Two negative edges emerged, between EAT-26 Social Pressure and EAI-3 Healthy Relevance and between EAT-26 Bulimia and Food Preoccupation and MEQ External Influences. EAT-26 Bulimia and Food Preoccupation showed the highest standardized strength, whereas MEQ Conscious Eating showed the strongest expected influence, and MEQ External Influences showed the highest betweenness. **Conclusions:** Exercise-addiction risk was selectively and modestly linked to eating-related components rather than to global disordered eating or mindful eating, with each instrument retaining substantial internal cohesion. The findings highlight the value of component-level Bayesian Gaussian network analysis for identifying candidate components associated with REA, with implications for screening and the design of future prospective and intervention studies. Because the EAI-3 subscales differ in valence, the Healthy Relevance dimension is interpreted as reflecting health-oriented exercise involvement rather than pathology. Finally, since the network is cross-sectional and undirected, the associations are non-causal, and generalizability is limited to recreational exerciser populations in Türkiye.

## 1. Introduction

Disordered eating encompasses a broad spectrum of eating-related problems, including dietary restriction, excessive food consumption, loss of control over food intake, and compensatory or purging behaviors such as excessive exercise, medication misuse, and self-induced vomiting [1]. Although these behaviors are not necessarily classified as clinical diagnoses, they may progress into clinically significant eating disorders if not identified and addressed early, highlighting the importance of prevention and early detection efforts [2].

The relationship between exercise addiction and eating-disorder symptoms has been consistently demonstrated by independent research groups and is considered a well-established research area [3,4,5]. Exercise addiction (EA) is conceptualized as a maladaptive behavioral pattern in which individuals lose control over their exercise behavior, leading to physical, psychological, or social harm [6,7,8]. Primary EA refers to a condition in which individuals engage in exercise for its inherent rewarding effects, making exercise an end in itself, whereas secondary EA is driven by body image concerns and weight-control motives, in which exercise serves to achieve external goals [9,10,11]. This primary–secondary distinction is theoretically important for the present study because the eating-related mechanisms examined here—dietary control, compensatory tendencies, and body- and shape-related concern—map most directly onto the weight- and shape-driven pathway; our analyses are primarily informative about secondary exercise-addiction risk, even though the EAI-3 indexes overall risk rather than separating the two subtypes. Individuals may develop EA to cope with emotions such as guilt and shame resulting from overeating, using exercise as a compensatory strategy to regulate eating behavior [12]. Therefore, eating disorder symptoms are considered among the key psychological processes associated with the risk of exercise addiction (REA).

Mindful eating involves paying attention to eating behavior without autopilot or emotional reactivity, engaging all senses, and recognizing internal cues such as hunger and satiety in a non-judgmental manner. It is therefore considered a protective mechanism against maladaptive eating patterns [13,14,15]. Research has consistently demonstrated a negative association between mindfulness and disordered eating tendencies [16,17,18,19,20]. Based on this evidence, mindfulness-based interventions have been shown to be effective in the prevention and treatment of eating disorders [21,22]. Mindful eating has been found to reduce disordered eating behaviors such as binge eating, emotional eating, and restrictive eating while enhancing self-regulation abilities and promoting a more balanced relationship with food [23,24,25,26,27,28,29].

Therefore, the use of exercise as a compensatory strategy for weight control and loss underscores the need to examine the relationship between compulsive exercise behaviors and secondary EA. Existing evidence suggests that mindful eating, disordered eating, and exercise addiction risk are interrelated; however, because the present design is cross-sectional and the estimated network is undirected, we make no assumption about the temporal or causal ordering of these associations and frame them throughout as conditional (synchronous) dependencies rather than pathways. In particular, understanding the role of psychological processes such as body image concerns, eating behaviors, and control needs, as well as the potential role of mindful eating in these processes, may contribute significantly to the EA literature.

Although the association between exercise-addiction risk and disordered eating is well established, prior evidence rests almost exclusively on correlational, regression, or latent-variable (structural-equation) models that treat each questionnaire as a single global score or as a manifestation of a single underlying common cause. Such approaches cannot reveal which specific components of eating behavior remain uniquely associated with exercise-addiction risk once all other components are held constant or identify the variables that occupy the most connective positions among these constructs. Bayesian Gaussian network analysis addresses this gap precisely: it models each subscale as a node and estimates the conditional (partial-correlation) dependency between each pair of nodes after adjusting for all remaining nodes, thereby distinguishing direct from spurious associations that global-score and regression models necessarily confound. The Bayesian estimator additionally quantifies the evidence for or against each edge through posterior inclusion probabilities (edge-evidence), providing a probabilistic safeguard against overinterpreting weak connections. Centrality and bridge indices derived from this network are not intended to imply causal primacy; rather, they indicate which components are most consistently interconnected (strength, expected influence) and which link otherwise separate clusters (betweenness, bridge expected influence). Such components are of interest clinically because they represent parsimonious, empirically prioritized candidates for screening and for future experimental or longitudinal testing—not confirmed intervention targets. Framing the study this way makes the analysis hypothesis-driven—asking where, and through which components, eating- and exercise-related constructs intersect—rather than merely method-driven.

Accordingly, the present study aims to examine the relationships among mindful eating, REA, and disordered eating tendencies in recreationally active individuals in Türkiye. Within this framework, the associations among the subdimensions of these variables were analyzed using Bayesian Gaussian network analysis to identify central and bridge variables in the network structure.

Consistent with component-level framing, we advanced four predictions. H1: Mindful eating components will show selective—rather than uniform—negative conditional associations with disordered eating components, with the strongest links involving awareness- and control-related facets. H2: Disordered eating components will be positively associated with exercise-addiction risk, but only for control- and compensation-related facets (e.g., dietary restriction, bulimia/food preoccupation) rather than for the disordered-eating construct as a whole. H3: Mindful eating will show differentiated associations with exercise-addiction risk such that awareness-oriented facets are protective, whereas rigidly disciplined/controlled facets are positively linked to risk. H4 (topological): control- and discipline-related eating components (e.g., MEQ Eating Discipline and EAT-26 Bulimia and Food Preoccupation) will emerge as central and bridging nodes connecting the eating and exercise domains.

## 2. Materials and Methods

### 2.1. Participants

In this cross-sectional study, participants included university students aged 18 years and older living in Türkiye who exercised at least 3 times per week, as well as voluntary recreational exercisers who regularly trained at fitness centers and sports clubs across Türkiye. The ≥3 sessions-per-week threshold functioned as an inclusion floor applied at the screening stage; consequently, the retained sample was truncated at three weekly sessions, and the mean of 4.40 sessions per week (SD = 1.51) reflects the expected right-skewed distribution of weekly frequency above this floor (most participants exercised three to six times weekly) rather than the retention of participants training fewer than three times per week. Using a snowball sampling method, participants who completed an online questionnaire via Google Forms (approximately 15–20 min) were recruited from various provinces between February and March 2026. Informed consent was obtained electronically through the online form. Data were collected anonymously. The researchers enrolled 303 individuals who met the inclusion criteria, aged between 18 and 57 years (M = 24.42, SD = 7.10), consisting of 57.8% men and 42.2% women (see Table 1). The study was approved by the Ethics Committee of Ağrı İbrahim Çeçen University (E-95531838-050.99-164648).

### 2.2. Measures

Mindful Eating Questionnaire (MEQ). The Mindful Eating Questionnaire (MEQ), developed by Framson et al. [30], was used to assess mindful eating and examine its core components, including disinhibition, awareness, external cues, distraction, and emotional response. The Turkish validity and reliability study of the scale was conducted by Kose et al. [31]. The scale is a 5-point Likert-type instrument consisting of 30 items and 7 subscales: Eating without Thinking, Emotional Eating, Eating Control, Mindfulness/Awareness, Eating Discipline, Conscious Eating, and External Influences. Items 1, 7, 9, 11, 13, 15, 18, 24, 25, and 27 are scored positively, while the remaining items are reverse-scored. Higher scores on each subscale indicate a stronger presence of the measured construct. Total mindful eating and subscale scores are calculated by averaging item scores; higher scores indicate greater mindful eating. The scale demonstrated good internal consistency, with a Cronbach’s alpha of 0.73 in the original study and 0.80 in the present study.

Eating Attitudes Test-26 (EAT-26). The Eating Attitudes Test-26 (EAT-26), developed by Garner et al. [32] and adapted into Turkish by Ergüney-Okumuş and Sertel-Berk [33], was used to assess tendencies toward eating disorders. The scale is a self-report, 6-point Likert-type instrument consisting of 26 items and three subscales: Restriction (Diet/Calorie Control), Bulimia and Food Preoccupation, and Social Pressure. Responses are scored as follows: Always = 3; Usually = 2; Often = 1; and Sometimes, Rarely, and Never = 0. Total scores range from 0 to 53 for the Turkish version, with higher scores indicating greater disordered eating attitudes. Cronbach’s alpha was 0.84 in the validation study and 0.87 in the present study.

Expanded Exercise Addiction Inventory, Third Version (EAI-3). The EAI-3, developed by Granziol et al. [11] and adapted into Turkish by Ozer et al. [34], was used to assess the risk of exercise addiction (REA). The scale is a 6-point Likert-type instrument consisting of 8 items and two subscales (Healthy Relevance and Addiction Tendency). Total scores range from 8 to 48. Because the two subscales differ in valence, they were interpreted separately throughout: Addiction Tendency indexes the symptom-like features of exercise dependence, whereas Healthy Relevance indexes the perceived importance and health-related salience of exercise and has been characterized in validation work as capturing comparatively healthy exercise involvement [11,34]. Higher total scores therefore reflect greater overall exercise-related involvement rather than pathology alone, and total scores are used here only for descriptive contextualization. Cronbach’s alpha was 0.81 in the validation study and 0.77 in the present study, indicating acceptable reliability. Consistent with the screening nature of this instrument, we refer throughout to exercise-addiction risk (REA) rather than to diagnosed exercise addiction.

### 2.3. Data Analysis

A Bayesian Gaussian graphical network analysis was conducted using 12 nodes representing all subscales of the EAT-26, MEQ, and EAI-3. Analyses were performed at the subscale level rather than the item level for three reasons: (a) the study’s questions concern relationships among theoretically defined facets (e.g., eating discipline, bulimia/food preoccupation, addiction tendency) rather than individual items; (b) a 12-node subscale network keeps the ratio of estimated parameters to observations within recommended bounds for stable estimation with *N* = 303, whereas a network of more than 60 items would be severely under-powered and unstable; and (c) subscale scores have established psychometric support, reducing the item-level measurement noise that can distort partial-correlation estimates. The analysis was estimated in JASP using a Gaussian graphical model estimator with 10,000 iterations and a burn-in of 5000. This approach estimates conditional dependencies among variables, represented as partial correlations after exerting control for all other nodes in the network. Edges therefore indicate unique associations between two variables conditional on the remaining variables, rather than causal or directional pathways.

The Bayesian Gaussian graphical model estimates an undirected network of partial correlations; it does not orient edges or yield a directed acyclic graph (DAG). Although JASP can also fit Bayesian DAGs, we deliberately used the undirected GGM because our aim was to characterize conditional dependencies (synchronous interrelationships) rather than to infer directional or causal pathways. Accordingly, all edges are symmetric and carry no arrowheads. Estimation used the default multivariate-normal likelihood with a conjugate prior over the precision matrix and a uniform prior over network structures (prior edge-inclusion probability = 0.5) so that the resulting edge-evidence (inclusion Bayes factors and posterior inclusion probabilities) reflects the data rather than an informative structural prior. Posterior sampling comprised 10,000 samples after a 5000-sample burn-in, and convergence was confirmed by inspecting the sampling behavior. The Gaussian assumption was examined by inspecting subscale distributions, and the network was additionally re-estimated after a nonparanormal (rank-based) transformation as a robustness check.

**Figure 1:**
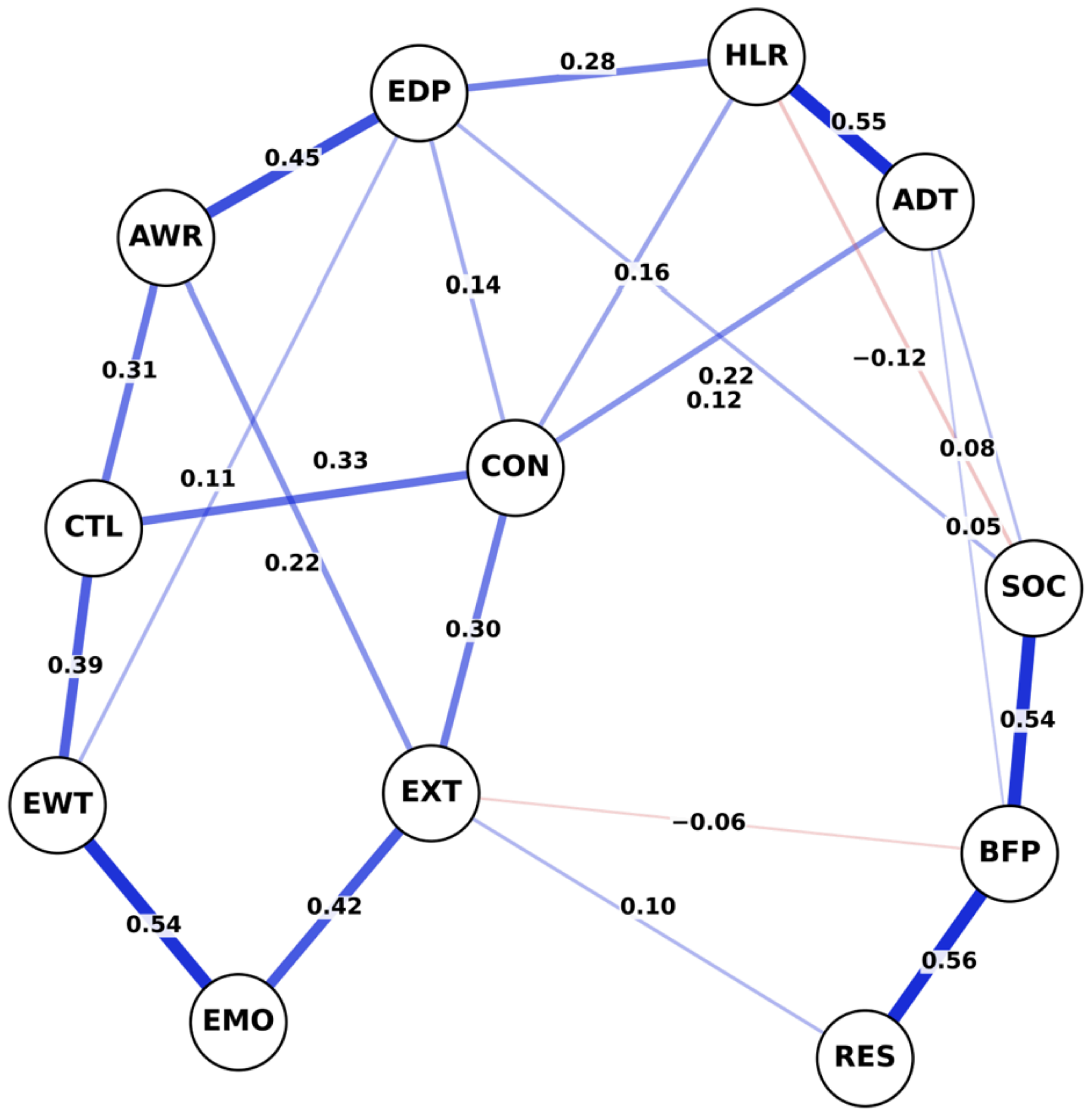
Estimated Bayesian Gaussian network among the subscales of the Eating Attitudes Test-26 (EAT-26), Mindful Eating Questionnaire (MEQ), and Expanded Exercise Addiction Inventory-3 (EAI-3). Edge width represents the magnitude of the conditional association (partial-correlation weight). Positive associations are shown as blue lines, whereas the two negative associations—between EAT-26 Social Pressure and EAI-3 Healthy Relevance (w = −0.116) and between EAT-26 Bulimia and Food Preoccupation and MEQ External Influences (w = −0.057)—are shown as red lines. Abbreviations: AWR = Mindfulness/Awareness; EDP = Eating Discipline; CON = Conscious Eating; CTL = Eating Control; EWT = Eating Without Thinking; EMO = Emotional Eating; EXT = External Influences; HLR = Healthy Relevance; ADT = Addiction Tendency; RES = Restriction (Diet/Calorie Control); BFP = Bulimia and Food Preoccupation; SOC = Social Pressure. A summary of all edges is provided in Table 2.

The estimated network contained 22 nonzero edges out of 66 possible edges, yielding a sparsity of 0.667. Centrality indices, including betweenness, closeness, strength, and expected influence, were computed to identify the most influential and connecting variables. Because posterior edge inclusion in a Bayesian graphical model is governed by edge-evidence Bayes factors rather than by resampling, case-dropping stability coefficients developed for regularized frequentist networks are not directly applicable; the robustness of the recovered structure was therefore examined in three ways. First, the network was re-estimated using a regularized frequentist Gaussian graphical model (EBICglasso, γ = 0.5) to establish whether the recovered edge structure depended on the choice of estimator. Second, age, sex, weekly exercise frequency, and weekly exercise duration were partialled out and the network re-estimated, and, in an additional model, these variables were entered as covariate nodes. Third, the network was re-estimated following a nonparanormal (rank-based) transformation to relax the assumption of multivariate normality. Finally, the prevalence of clinically relevant elevations was computed using the conventional EAT-26 cut-off of ≥20 and an equivalent upper-range threshold on the EAI-3 (≥34) to contextualize the sample. The full weight matrix and centrality indices are reported in the Supplementary Materials (Tables S2 and S3). The network is illustrated in Figure 1 and Table 2.

## 3. Results

### 3.1. Network Structure

The network consisted of 12 variables and 22 nonzero edges among 66 possible connections, yielding a sparsity of 0.667. This indicates a moderately sparse structure in which approximately one-third of all possible conditional associations were retained. For context, 19.5% (*n* = 59) of participants scored at or above the conventional EAT-26 screening cut-off of 20, indicating elevated disordered-eating attitudes, and 33.4% (*n* = 101) scored at or above the corresponding EAI-3 threshold, situating the present sample within a predominantly subclinical range. Because the EAI-3 total combines the Addiction Tendency and Healthy Relevance subscales, the latter figure indexes overall exercise-related involvement rather than exercise addiction as such. Overall, the network indicated that the three constructs retained substantial internal cohesion, with REA connected to eating-related components through a small number of specific and comparatively weak conditional associations rather than through a broad link to disordered eating as a unified construct.

The strongest conditional associations in the network occurred within rather than between instruments. Within the EAT-26, Restriction (Diet/Calorie Control) was strongly associated with Bulimia and Food Preoccupation (w = 0.560), which was in turn associated with Social Pressure (w = 0.537); the zero-order association between Restriction and Social Pressure was accounted for by these paths and did not survive as an edge. Within the EAI-3, Healthy Relevance and Addiction Tendency were strongly linked (w = 0.552). Within the MEQ, a densely connected cluster emerged, anchored by Eating without Thinking–Emotional Eating (w = 0.539), Mindfulness/Awareness–Eating Discipline (w = 0.447), Emotional Eating–External Influences (w = 0.416), and Eating without Thinking–Eating Control (w = 0.393). Two negative edges were retained, between EAT-26 Social Pressure and EAI-3 Healthy Relevance (w = −0.116) and between EAT-26 Bulimia and Food Preoccupation and MEQ External Influences (w = −0.057), indicating that higher socially driven eating concern was conditionally associated with lower endorsement of exercise as a healthy and important life component.

Cross-domain associations were present but consistently weaker than within-instrument associations. The mindful-eating and exercise-addiction domains were bridged primarily by MEQ Eating Discipline–EAI-3 Healthy Relevance (w = 0.282), MEQ Conscious Eating–EAI-3 Addiction Tendency (w = 0.216), and MEQ Conscious Eating–EAI-3 Healthy Relevance (w = 0.163). Associations between the disordered-eating and exercise-addiction domains were weaker still, comprising EAT-26 Social Pressure–EAI-3 Healthy Relevance (w = −0.116), EAT-26 Social Pressure–EAI-3 Addiction Tendency (w = 0.084), and EAT-26 Bulimia and Food Preoccupation–EAI-3 Addiction Tendency (w = 0.053). Links between the disordered-eating and mindful-eating domains followed the same pattern, comprising EAT-26 Social Pressure–MEQ Eating Discipline (w = 0.121), EAT-26 Restriction–MEQ External Influences (w = 0.101), and EAT-26 Bulimia and Food Preoccupation–MEQ External Influences (w = −0.057). Notably, no direct association emerged between EAI-3 Addiction Tendency and MEQ Eating Control once the remaining nodes were taken into account. These results indicate that exercise-addiction risk is connected to eating-related processes through conscious and disciplined eating rather than through eating control as such, and that these connections are modest in magnitude.

### 3.2. Centrality Analyses

Centrality analyses identified several nodes with comparatively high connectivity. EAT-26 Bulimia and Food Preoccupation showed the highest standardized strength (z = 1.25), followed by MEQ Conscious Eating (z = 0.89), EAI-3 Healthy Relevance (z = 0.62), and MEQ Eating Discipline (z = 0.55). Expected influence, which retains the edge sign, was highest for MEQ Conscious Eating (z = 1.29), followed by MEQ Eating Discipline (z = 0.92) and EAT-26 Bulimia and Food Preoccupation (z = 0.84).

Betweenness centrality identified MEQ External Influences (z = 1.50), MEQ Conscious Eating (z = 0.69), and MEQ Eating Discipline (z = 0.58) as the principal bridging variables. Consistent with the edge-level results, the mindful-eating subscales carried most of the cross-domain connectivity in the network, whereas the EAI-3 subscales occupied comparatively peripheral positions (Addiction Tendency z = −0.74; Healthy Relevance z = −0.42). Overall, the findings suggest that REA-related processes are connected to a limited set of eating-related components rather than being embedded within a generalized disordered-eating structure.

### 3.3. Network Accuracy and Stability

To assess whether the recovered structure depended on the choice of estimator, the network was re-estimated using a regularized frequentist Gaussian graphical model. The two solutions agreed closely at the edge level: edge weights correlated r = 0.819 across all 66 possible connections, 75.8% of connections were classified identically as present or absent, and the four strongest edges were identical under both estimators (Table S4). Centrality indices, however, proved considerably less robust to estimator choice, with low rank-order agreement between the two solutions for both strength (ρ = −0.08) and expected influence (ρ = −0.18); this reflects the fact that regularization removed a set of moderate mindful-eating edges that the Bayesian model retained, which in turn redistributed node connectivity. The edge structure reported above should therefore be regarded as substantially better supported than the centrality ordering, and the centrality findings are interpreted here as descriptive of the present solution rather than as stable estimates of node importance.

### 3.4. Sensitivity Analyses

When age, sex, weekly exercise frequency, and weekly exercise duration were partialled out and, in a separate model, entered as covariate nodes, the overall network structure remained materially unchanged, with edge weights showing correlations of r = 0.993 and r = 0.999 with the unadjusted solution, respectively, indicating that the reported associations are unlikely to be artifacts of demographic composition. Only weak covariate edges were retained, the strongest being age–MEQ Eating Discipline (w = 0.104) and age–MEQ Eating Control (w = 0.071). The network re-estimated after a nonparanormal (rank-based) transformation was likewise highly consistent with the primary solution (r = 0.978). These robustness analyses were conducted using the regularized frequentist estimator and are reported in full in the Supplementary Materials (Table S4).

## 4. Discussion

This study aimed to examine the relationships among mindful eating, disordered eating tendencies, and REA in recreational exercisers. The Bayesian Gaussian network analysis revealed a moderately sparse network structure consisting of 12 nodes and 22 nonzero edges. The findings indicated that REA was associated with specific dimensions across both disordered eating and mindful eating constructs but that these cross-domain associations were considerably weaker than the associations observed within each instrument. Although the relationship between REA and disordered eating symptoms has been well documented in the literature [3,4,5,35,36,37,38], this study contributes novel evidence by integrating EAT-26 subscales, mindful eating dimensions, and EAI-3 subdimensions within a single Bayesian Gaussian network framework. Overall, the findings provided partial support for the hypotheses: mindful eating components showed selective, rather than uniformly negative, associations with disordered eating and EAI-3 dimensions, while disordered eating components were only weakly linked to exercise-addiction-risk-related nodes.

Two interpretive caveats frame the discussion that follows. First, throughout we refer to exercise-addiction risk (REA), because the EAI-3 is a screening instrument indexing risk and symptomatology rather than a diagnosed disorder; the shorter term REA denotes the risk construct. Second, because the centrality and edge estimates derive from a cross-sectional, undirected partial-correlation network, they identify statistically interconnected components—not causal drivers, therapeutic mechanisms, or temporally prior variables. High centrality does not establish that intervening on a node will change the others. The interpretive and intervention-related statements below should therefore be read as hypotheses for future experimental and longitudinal testing rather than as demonstrated mechanisms or established targets.

Overall, the findings demonstrated that REA was connected to eating-related processes through a small number of specific and comparatively weak conditional associations. The Addiction Tendency subscale of the EAI-3 was linked most clearly to MEQ Conscious Eating and more weakly to EAT-26 Social Pressure and Bulimia and Food Preoccupation, whereas no direct association with eating control emerged once the remaining nodes were taken into account. These results suggest that REA is not broadly related to disordered eating pathology and that its association with eating regulation is narrower than zero-order correlational work has implied. That the strongest associations in the network occurred within rather than between instruments is itself informative: at the component level, mindful eating, disordered eating attitudes, and exercise-addiction risk behaved as internally cohesive but only loosely coupled systems.

In contrast to this pattern, the four predictions received mixed support. H1, which anticipated selective negative conditional associations between mindful eating and disordered eating components, was only partially supported: a single weak negative edge emerged (Bulimia and Food Preoccupation–External Influences, w = −0.057), alongside two weak positive links. H2, which predicted positive associations between control- and compensation-related disordered eating facets and exercise-addiction risk, was supported in direction but not in magnitude, with both relevant edges below w = 0.09. H3, which predicted that awareness-oriented mindful eating facets would be protective while disciplined facets would be positively linked to risk, was not supported as stated: no negative mindful-eating–exercise-addiction edges emerged, and the observed links involved conscious and disciplined eating in the positive direction. H4 received partial support in that Bulimia and Food Preoccupation was the strongest node and Eating Discipline occupied a bridging position, although the highest betweenness value belonged to External Influences. Importantly, these associations admit more than one interpretation. In recreational exercisers, the link between conscious eating and addiction tendency may equally reflect adaptive self-regulation—deliberate attention to nutrition that supports training goals—rather than incipient pathology. A cross-sectional network cannot distinguish adaptive from maladaptive self-regulation; the same attentional facet may be protective at moderate levels and risk-related only when it becomes rigid and inflexible. We therefore interpret the Conscious Eating and Eating Discipline associations as markers of a deliberate, control-oriented behavioral style whose clinical significance is context-dependent, rather than as inherently maladaptive processes.

The strongest association in the network, between EAT-26 Restriction (Diet/Calorie Control) and EAT-26 Bulimia and Food Preoccupation (w = 0.560), suggests that restrictive eating and bulimic-type symptoms are closely linked within the disordered eating domain. This finding is consistent with the literature suggesting that restrictive eating is often followed by binge-type eating and compensatory behaviors [39,40,41,42]. Alternatively, individuals may experience perceived loss of control following dietary restraint, which may be associated with feelings of guilt and anxiety and subsequent bulimic or compensatory behaviors [43,44]. That the direct Restriction–Social Pressure association did not survive conditioning suggests that bulimic-type preoccupation may account for the observed link between dietary restraint and socially driven eating concern.

Outside its own instrument, the Healthy Relevance dimension of the EAI-3 was most strongly associated with MEQ Eating Discipline (w = 0.282) and MEQ Conscious Eating (w = 0.163), and negatively with EAT-26 Social Pressure (w = −0.116). This pattern suggests that perceiving exercise as healthy and important is embedded in a broader self-regulatory system involving deliberate and disciplined eating and is conditionally lower among those reporting greater socially driven eating concern. Considerable interpretive caution is required at this point. Unlike Addiction Tendency, Healthy Relevance has been characterized in validation work as capturing comparatively healthy and committed exercise involvement rather than a pathological process [11,34]. Its position within the present network should therefore not be read as evidence that involvement in health-oriented exercise is itself a risk process. Its associations with disciplined and conscious eating are more parsimoniously interpreted as reflecting a shared and broadly adaptive self-regulatory style, and its negative association with Social Pressure is consistent with that reading. We accordingly refrain from interpreting this node in pathological terms: the inclusion of both EAI-3 subscales as nodes reflects the structure of the instrument and should not be taken to imply that both index risk of exercise addiction. The magnitude of these associations was, in any case, modest and warrants replication.

Centrality analyses highlighted EAT-26 Bulimia and Food Preoccupation, MEQ Conscious Eating, and MEQ Eating Discipline as comparatively well-connected variables. Bulimia and Food Preoccupation showed the highest strength, indicating strong overall connectivity with other nodes. The pattern indicates that bulimic-type symptoms are the most densely interconnected components and may therefore be statistically well placed to link disordered eating patterns with exercise-related processes. This interpretation is supported by previous research showing that individuals engaging in compensatory exercise also tend to report elevated bulimic symptoms [45,46,47]. As noted in Section 3.3, however, the centrality ordering proved sensitive to the choice of estimator, and these node-level interpretations should accordingly be treated as provisional. Importantly, the edge-level findings were substantially more robust than the centrality rankings across estimators; therefore, greater confidence should be placed in the presence and magnitude of the reported associations than in the precise ordering of node importance.

MEQ Conscious Eating showed the highest expected influence and MEQ External Influences the highest betweenness, indicating that the mindful-eating subscales occupied the network’s principal bridging positions. Theoretically, the prominence of conscious and disciplined eating is broadly consistent with dual-pathway and restraint models, in which sustained deliberate control over eating operates as a common upstream regulatory demand that both constrains eating behavior and feeds the rule-governed, achievement-oriented cognition characteristic of compulsive exercise. On this reading, deliberate eating regulation may act as a hinge between two otherwise loosely coupled behavioral systems, which would help explain why the cross-domain edges that did emerge ran predominantly through conscious and disciplined eating rather than through disordered eating symptoms directly. This interpretation is consistent with restraint theory and cognitive models of eating regulation, which propose that sustained dietary control may initially serve as a self-regulatory strategy but can become cognitively and emotionally demanding over time [48,49,50]. It remains to be tested longitudinally.

By contrast, the EAI-3 subscales occupied comparatively peripheral positions in the network, with below-average betweenness values. Exercise-related processes were therefore not isolated from eating-related components, but neither did they function as integrating hubs; rather, they were attached to the eating-related system through a small number of specific links involving conscious and disciplined eating. From a network perspective, it is the mindful-eating components that appear to integrate the different behavioral domains within a broader self-regulatory system [51,52].

Overall, the findings suggest a broader self-regulatory framework in which eating behaviors, mindful eating processes, and tendencies toward REA are interconnected. More rigid, control-oriented patterns may be associated with maladaptive outcomes, whereas conscious, awareness-based processes may reflect more adaptive regulation.

Finally, these findings suggest that interventions targeting REA may benefit from considering not only exercise behavior but also underlying eating behavior processes. In particular, eating control, eating discipline, and bulimic symptoms may be candidate targets that warrant prospective and experimental evaluation before being adopted in practice. Furthermore, mindfulness-based eating interventions may play a protective role by supporting more adaptive and less automatic eating regulation [16,53,54,55].

From a practical standpoint, and contingent on prospective confirmation, the component-level pattern points to concrete, tiered applications. For screening, if replicated in independent samples, brief tools targeting the most salient components—bulimia/food preoccupation and disciplined or controlled eating—may more efficiently flag recreational exercisers at elevated risk than global questionnaires and could be embedded in routine intake at fitness centers and university sports services. For prevention, psychoeducation in these settings could specifically address the restriction–bulimia link and the tendency to convert rigid eating rules into rigid exercise rules while normalizing flexible, goal-appropriate nutrition. For intervention, mindfulness-based eating programs that strengthen awareness and conscious-eating facets (which appeared adaptive here) while loosening rigid dietary control offer a theoretically coherent and testable approach for individuals already showing elevated exercise-addiction risk. Because these implications rest on cross-sectional associations, they are best regarded as a prioritized research agenda—candidate screening items and intervention foci to be evaluated in controlled prospective designs—rather than as ready-made clinical recommendations.

## 5. Limitations

The study employed a cross-sectional design; therefore, causal inferences cannot be made, and the edges in the Bayesian Gaussian network should not be interpreted as causal or directional pathways. Relatedly, the centrality and bridge indices are derived from an undirected, cross-sectional network and therefore index statistical interconnection rather than causal or therapeutic importance; they should not be read as evidence that any single component drives the others. A related caution applies to the EAI-3, whose two subscales differ in valence: Healthy Relevance and Addiction Tendency appear side by side as nodes because they belong to the same instrument, and their adjacency does not imply that both index pathological processes. The Bayesian Gaussian network analysis is inherently exploratory; its results should be treated as hypothesis-generating rather than confirmatory. The data were based on self-report measures, which may introduce potential response and social desirability biases.

Several potentially confounding or explanatory variables were not assessed and could not be incorporated into the network, including body mass index, the type and modality of sport, competitive versus purely recreational status, exercise motivation, personal or family history of eating disorders or other psychiatric diagnoses, and nutritional status. Their omission constrains interpretation, because some observed associations may be shaped by these unmeasured characteristics; future studies should measure and model them explicitly. Although age, sex, and exercise frequency and duration were examined in sensitivity analyses (Section 3.4), these covariates capture only part of the relevant heterogeneity.

The sample was drawn exclusively from Türkiye using a non-probability snowball sampling method, which limits the representativeness and cross-cultural generalizability of the findings. The sample was predominantly composed of young adults (M = 24.42 years), with a large proportion being university students, which may not reflect the broader recreational exerciser population. These features warrant particular caution: convenience recruitment from a single, predominantly young and student-heavy, Turkish sample means the findings may not generalize to older recreational exercisers, to other cultural or linguistic contexts, or—importantly—to elite and competitive athletes, in whom disciplined eating and high training loads carry different normative meanings and may relate to exercise-addiction risk differently. All measures were subclinical self-report instruments; no clinical diagnoses of eating disorders or REA were confirmed, and therefore, the findings pertain to tendencies and risk rather than clinical conditions. Future studies may benefit from longitudinal designs to examine how these relationships evolve over time. In addition, comparative network analyses across different athlete groups and cultural contexts are recommended to further explore the robustness and generalizability of these findings. Specifically, we recommend replication in independent, larger, and more demographically diverse samples—including elite athletes and clinical populations—and, critically, longitudinal and experimental designs capable of testing the directional and intervention-related hypotheses that the present network can only generate.

## 6. Conclusions

In conclusion, this study demonstrated that REA processes are connected to a limited and specific set of eating-related components rather than to a generalized disordered eating structure. Bayesian Gaussian network analysis highlighted EAT-26 Bulimia and Food Preoccupation, MEQ Conscious Eating, and MEQ Eating Discipline as the most connected variables within the network. Specifically, EAT-26 Bulimia and Food Preoccupation showed the highest standardized strength, MEQ Conscious Eating the strongest expected influence, and MEQ External Influences the highest betweenness. These findings indicate that bulimic-type symptoms and deliberate eating regulation may be especially important in statistically connecting eating-related and exercise-related processes, although the comparatively weak cross-domain edges indicate that this connection is selective rather than pervasive.

Overall, the findings indicate that REA is associated with deliberate, control-oriented eating processes rather than with global eating disorder symptoms and that these associations are modest in magnitude. Mindful eating components appear to play differentiated roles: conscious eating and eating discipline carried most of the cross-domain connectivity, whereas the remaining mindful-eating facets were confined largely to within-instrument associations.

These results suggest that interventions targeting REA may benefit from adopting a broader perspective that includes not only exercise behavior but also underlying eating-related cognitive and emotional processes. Reducing rigid dietary control and addressing bulimic symptoms may be important candidate targets warranting prospective confirmation, while mindfulness-based approaches may offer a protective function by enhancing adaptive self-regulation. Future research should employ prospective designs and multi-sample replication to confirm the stability of these network structures and evaluate the efficacy of integrated mindfulness and eating-regulation interventions in reducing exercise-addiction risk among recreational populations.

## Figures and Tables

**Table 1:** Participants’ demographic characteristics (*N* = 303).

Variable	Category	*n*	%	M (SD)
Gender	Men	175	57.8	
	Women	128	42.2	
Age (years)				24.42 (7.10)
Exercise frequency (per week)				4.40 (1.51)
Exercise duration (hours/week)				7.13 (6.95)
Total (valid)		303	100	

Note. M = mean; SD = standard deviation. Data were collected between February and March 2026 via snowball sampling from various provinces in Türkiye.

**Table 2:** Principal conditional associations (edges) in the estimated network.

Edge (Node 1—Node 2)	Weight (w)	Domains Linked
EAT-26 Restriction (Diet/Calorie Control)—EAT-26 Bulimia and Food Preoccupation	0.560	ED—ED
EAI-3 Healthy Relevance—EAI-3 Addiction Tendency	0.552	EA—EA
MEQ Eating without Thinking—MEQ Emotional Eating	0.539	ME—ME
EAT-26 Bulimia and Food Preoccupation—EAT-26 Social Pressure	0.537	ED—ED
MEQ Mindfulness/Awareness—MEQ Eating Discipline	0.447	ME—ME
MEQ Emotional Eating—MEQ External Influences	0.416	ME—ME
MEQ Eating without Thinking—MEQ Eating Control	0.393	ME—ME
MEQ Eating Control—MEQ Conscious Eating	0.329	ME—ME
MEQ Eating Control—MEQ Mindfulness/Awareness	0.309	ME—ME
MEQ Conscious Eating—MEQ External Influences	0.297	ME—ME
MEQ Eating Discipline—EAI-3 Healthy Relevance	0.282	ME—EA
MEQ Conscious Eating—EAI-3 Addiction Tendency	0.216	ME—EA
MEQ Mindfulness/Awareness—MEQ External Influences	0.216	ME—ME
MEQ Conscious Eating—EAI-3 Healthy Relevance	0.163	ME—EA
MEQ Eating Discipline—MEQ Conscious Eating	0.141	ME—ME
EAT-26 Social Pressure—MEQ Eating Discipline	0.121	ED—ME
EAT-26 Social Pressure—EAI-3 Healthy Relevance	−0.116	ED—EA
MEQ Eating without Thinking—MEQ Eating Discipline	0.114	ME—ME
EAT-26 Restriction (Diet/Calorie Control)—MEQ External Influences	0.101	ED—ME
EAT-26 Social Pressure—EAI-3 Addiction Tendency	0.084	ED—EA
EAT-26 Bulimia and Food Preoccupation—MEQ External Influences	−0.057	ED—ME
EAT-26 Bulimia and Food Preoccupation—EAI-3 Addiction Tendency	0.053	ED—EA

Note. Edges are listed by descending absolute weight; w = partial-correlation edge weight. ED = disordered-eating domain (EAT-26); ME = mindful-eating domain (MEQ); EA = exercise-addiction-risk domain (EAI-3). The complete 22-edge weight matrix is provided in the Supplementary Materials (Table S2).

## Data Availability

All data are stored on the corresponding author’s institutional servers and are available upon reasonable request.

## Supplementary Materials

The following supporting information can be downloaded at: https://www.mdpi.com/article/10.3390/nu18152524/s1, Table S1: Summary of Bayesian Network. Table S2: Bayesian Network Weights Matrix. Table S3: Centrality Measures per Variable. Table S4: Robustness and Sensitivity Analyses.
