# Supplementary material for: Mindful Eating, Disordered Eating, and Exercise Addiction: A Bayesian Gaussian Network Analysis
Source: Nutrients. 2026 Aug 4;18(15):2524. doi: 10.3390/nu18152524 (PMC13468564; doi:10.3390/nu18152524)
Supplement: Supplementary file 1 [file nutrients-18-02524-s001.zip › nutrients-4434154-supplementary.pdf]

Supplementary Materials

Mindful Eating, Disordered Eating, and Exercise Addiction: A Bayesian Gaussian Network Analysis

Table S1. Summary of Bayesian Network.

Number of Nodes	Number of Non-Zero Edges	Sparsity
12	22 / 66	0.667

Note. Sparsity = proportion of zero edges = 1 – (22/66) = .667. Analysis estimated using a Gaussian graphical model (GGM estimator, g-prior = 0.5) with 10,000 MCMC iterations and burn-in of 5,000.

Table S2. Bayesian Network Weights Matrix.

Variable	EAT-R	EAT-B	EAT-SP	MEQ-EwT	MEQ-EE	MEQ-EC	MEQ-MA	MEQ-ED	MEQ-CE	MEQ-EI	EAI-HR	EAI-AT
**EAT-26 Restriction**	—	**0.560**	—	—	—	—	—	—	—	0.101	—	—
**EAT-26 Bulimia**	**0.560**	—	**0.537**	—	—	—	—	—	—	-0.057	—	0.053
**EAT-26 Social Pressure**	—	**0.537**	—	—	—	—	—	**0.121**	—	—	- 0.116	0.084
**MEQ Eating without Thinking**	—	—	—	—	0.539	0.393	—	0.114	—	—	—	—
**MEQ Emotional Eating**	—	—	—	0.539	—	—	—	—	—	0.416	—	—
**MEQ Eating Control**	—	—	—	0.393	—	—	**0.309**	—	0.329	—	—	—
**MEQ Mindfulness**	—	—	—	—	—	**0.309**	—	0.447	—	**0.216**	—	—
**MEQ Eating Discipline**	—	—	**0.121**	0.114	—	—	0.447	—	0.141	—	0.282	—
**MEQ Conscious Eating**	—	—	—	—	—	0.329	—	0.141	—	0.297	**0.163**	**0.216**
**MEQ External Influences**	0.101	-0.057	—	—	0.416	—	**0.216**	—	0.297	—	—	—
**EAI-3 Healthy Relevance**	—	—	-0.116	—	—	—	—	0.282	**0.163**	—	—	0.552
**EAI-3 Addiction Tendency**	—	0.053	0.084	—	—	—	—	—	**0.216**	—	0.552	—

Note. Abbreviations: EAT-R = EAT-26 Restriction (Diet/Calorie Control); EAT-B = EAT-26 Bulimia and Food Preoccupation; EAT-SP = EAT-26 Social Pressure; MEQ-EwT = MEQ Eating without Thinking; MEQ-EE = MEQ Emotional Eating; MEQ-EC = MEQ Eating Control; MEQ-MA = MEQ Mindfulness/Awareness; MEQ-ED = MEQ Eating Discipline; MEQ-CE = MEQ Conscious Eating; MEQ-EI = MEQ External Influences; EAI-HR = EAI-3 Healthy Relevance; EAI-AT = EAI-3 Addiction Tendency. Non-zero edges are highlighted (green = positive; red = negative). Dashes (—) indicate zero edges (absent conditional associations).

Table S3. Centrality Measures per Variable.

Variable	Betweenness	Closeness	Strength	Expected Influence
**EAT-26 Restriction**	-0.448	-1.488	-1.870	-1.470
**EAT-26 Bulimia**	0.173	-1.349	1.252	0.841
**EAT-26 Social Pressure**	0.245	-1.207	-0.570	-1.765
**MEQ Eating without Thinking**	-0.984	-0.244	0.177	0.589
**MEQ Emotional Eating**	-0.694	-0.231	-0.439	-0.055
**MEQ Eating Control**	0.009	0.291	0.069	0.479
**MEQ Mindfulness/Awareness**	0.078	0.847	-0.346	0.072
**MEQ Eating Discipline**	0.580	1.007	0.553	0.921
**MEQ Conscious Eating**	0.694	0.903	0.889	1.288
**MEQ External Influences**	1.503	0.921	0.464	0.060
**EAI-3 Healthy Relevance**	-0.415	0.330	0.619	-0.518
**EAI-3 Addiction Tendency**	-0.741	0.221	-0.797	-0.441

Note. Centrality indices are standardized z-scores. Betweenness = number of shortest paths passing through a node; Closeness = inverse of average shortest path distance; Strength = sum of absolute edge weights; Expected Influence = sum of signed edge weights. All indices derived from the Bayesian network weight matrix.

Table S4. Robustness and Sensitivity Analyses.

Model	Edge-weight r	Edge agreement
Regularized frequentist counterpart (EBICglasso, γ = 0.5)	.819	75.8%
Covariates partialled out (age, sex, exercise frequency, duration)	.993	92.4%
Covariates entered as nodes	.999	98.5%
Nonparanormal (rank-based) transformation	.978	—

Note. Row 1 compares the regularized frequentist solution with the primary Bayesian solution reported in Table S2; rows 2–4 compare each adjusted frequentist model with the unadjusted frequentist solution. Edge agreement = percentage of the 66 possible connections classified identically as present or absent. Covariate-adjusted models were estimated on N = 291 complete cases. Covariate edges retained in the covariates-as-nodes model were uniformly weak: age–MEQ Eating Discipline (.104), age–MEQ Eating Control (.071), sex–MEQ Emotional Eating (−.090), sex–EAI-3 Addiction Tendency (−.023). Rank-order agreement in centrality between the Bayesian solution and its frequentist counterpart was low (strength ρ = −.08; expected influence ρ = −.18) and is discussed in Section 3.3 of the main text.
